# The average does not represent the individual: White matter variability across the brain, across the population, and across the lifespan

**DOI:** 10.21203/rs.3.rs-8654078/v1

**Published:** 2026-01-29

**Authors:** Kurt G Schilling, Lilit Dulyan, Eva Guzmán Chacón, Matthew Amandola, Michael Kim, Bennett A Landman, Stephanie J. Fokel

**Affiliations:** Vanderbilt University Institute of Imaging Science, Vanderbilt University Medical Center, Nashville, TN, United States; Department of Radiology and Radiological Sciences, Vanderbilt University Medical Center, Nashville, TN, United States; Donders Institute for Brain Cognition and Behaviour, Radboud University, Nijmegen, The Netherlands; Brain Connectivity and Behaviour Laboratory, Sorbonne Universities, Paris, France; Max Planck Institute for Psycholinguistics, Nijmegen, The Netherlands; Donders Institute for Brain Cognition and Behaviour, Radboud University, Nijmegen, The Netherlands; Vanderbilt University Institute of Imaging Science, Vanderbilt University Medical Center, Nashville, TN, United States; Department of Radiology and Radiological Sciences, Vanderbilt University Medical Center, Nashville, TN, United States; Department of Biomedical Engineering, Vanderbilt University, Nashville, TN, United States; Department of Electrical Engineering and Computer Engineering, Vanderbilt University, Nashville, TN, USA; Vanderbilt University Institute of Imaging Science, Vanderbilt University Medical Center, Nashville, TN, United States; Department of Radiology and Radiological Sciences, Vanderbilt University Medical Center, Nashville, TN, United States; Department of Biomedical Engineering, Vanderbilt University, Nashville, TN, United States; Department of Electrical Engineering and Computer Engineering, Vanderbilt University, Nashville, TN, USA; Donders Institute for Brain Cognition and Behaviour, Radboud University, Nijmegen, The Netherlands; Brain Connectivity and Behaviour Laboratory, Sorbonne Universities, Paris, France; Max Planck Institute for Psycholinguistics, Nijmegen, The Netherlands

**Keywords:** neurovariability, white matter, development, aging, cognition

## Abstract

Inter-individual differences in brain anatomy are often treated as noise, yet may encode meaningful principles of organisation. Using >2,800 diffusion MRI scans spanning 0–100 years, we quantified spatial, microstructural and macrostructural variability across 64 major white-matter pathways. Variability is not random: spatial organisation follows a deep-to-superficial gradient, and variability across individuals shows a convergence–divergence profile, decreasing in early development and increasing in ageing. Microstructural and macrostructural features exhibit distinct trajectories and hemispheric asymmetries. Importantly, tract variability relates to individual differences in behaviour, most strongly during development. These results redefine white-matter variability as a structured, developmentally dynamic, lifespan-dependent feature of brain organisation and provide a foundation for normative modelling and precision neuroscience.

## Introduction

The human brain is anatomically diverse [1, 2], yet much of systems neuroscience remains grounded in group averages. White matter is a major source of this diversity: axonal pathways connect distributed regions into large-scale networks that support cognition and behaviour [3]. Although white-matter properties have been linked to functional differences across individuals and clinical groups [4–6], we still lack a systematic account of normative white-matter variability [7]. That is, how it is organised across pathways, how it changes across the lifespan, and when it is functionally meaningful.

Variability may occur both *across the brain* (intraindividual variability across different white matter pathways/hemispheres) and *across the population* (interindividual variability between people), and these variations may change *across the lifespan* due to healthy developmental, healthy aging, and pathological patterns (lifespan variability) [1, 8, 9]. Differences across pathways have long been recognized, where macroscopic variations in shape, location, and trajectory are used to describe and classify various long-range association, projection, and commissural pathways [8, 10–12]. Inter-individual variability serves as a foundation for many white matter neuroimaging studies [3, 13], where differences in cognitive or behavioral performance across and between healthy or clinical populations are used to identify functional associations with specific white matter pathways [14, 15]. In parallel, white matter properties are known to change across the lifespan [16, 17], with distinct rates and patterns across developmental periods and ageing [14, 18–21].

White-matter variability is not confined to a single anatomical scale but emerges across multiple, partially independent features. At the spatial level, pathways vary in their location and trajectory across individuals. Such differences can be appreciated in postmortem dissections, where variability in pathway terminations drive differences in surface anatomy and the apparent position of tract endpoints, and differences in gross anatomy shift the relative locations of dissected bundles [22]. At the microstructural level, pathways differ in neurite densities, diameters, myelination, and orientation dispersion, both across tracts and across individuals [23–28]. These properties can be quantified in vivo using advanced neuroimaging techniques [29–32], most notably diffusion magnetic resonance imaging (dMRI), which is exquisitely sensitive to the underlying tissue microstructure [30]. Finally, variation exists in the macroscopic features of these pathways[16, 33]. These include geometrical and shape descriptors of volumes, areas, and lengths derived from diffusion MRI fiber tractography, which have been shown to also vary across the brain, across the population, and across the lifespan.

Recent literature recognises structural variability in white matter as a critical neurobiological substrate underlying cognitive and behavioural variability [1, 34]. Contrary to perspectives that may view anatomical variability primarily as methodological noise or a confound to group-level neuroimaging analyses, studies increasingly demonstrate that structural differences meaningfully explain variability in cognition and behaviour. Variations in the organisation and microstructure of white matter tracts have been linked to differences in language processing, executive function, and memory performance [3]. Structural variations, including hemispheric asymmetries, reflect developmental and evolutionary specialization [35] and are closely associated with cognitive and motor functions, such as language dominance, motor skills, and attentional control [36, 37]. Importantly, most cognitive and behavioral functions rely on multiple, interconnected white matter pathways [3]; consequently, structural variability in even a single pathway can influence multiple functional domains simultaneously. Despite these insights, comprehensive, pathway-specific assessments of structural variability and their broader associations with cognitive and behavioral traits across the human lifespan remain limited.

In this study, we pool diffusion MRI data from 2,854 participants to provide a comprehensive, multi-scale characterisation of normative white-matter variability. Using high-quality tractography, we quantify variability across spatial (e.g. pathway topography), microstructural (e.g. fractional anisotropy), and macrostructural (e.g. tract volume) features in 64 long-range association, projection, and commissural pathways. The cohort spans the full human lifespan, from infancy to late adulthood (0–100 years), enabling systematic assessment of how white-matter variability differs across pathways, across individuals, and across developmental and ageing stages. We further examine how variability in specific pathways and structural features relates to inter-individual differences in cognitive, motor, sensory, emotional, and personality traits. Together, this work establishes a normative, lifespan-informed framework for white-matter variability and directly links multi-scale anatomical diversity to meaningful behavioural differences across individuals.

## Results

**Figure 1:**
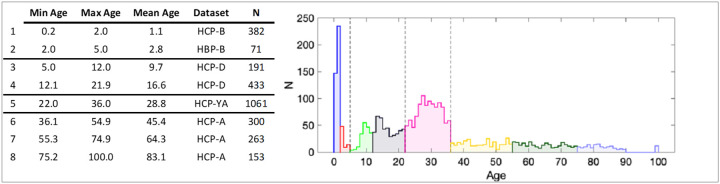
Datasets, age ranges, and cohorts across the lifespan.

To characterise white matter variability across the human lifespan, we leveraged high-quality diffusion MRI datasets spanning postnatal infancy to late adulthood (0–100 years), drawn from multiple branches of the Human Connectome Project [38, 39] (Figure 1). Using state-of-the-art image processing pipelines [40] and automated tractography [41], we reconstructed 64 long-range association, projection, commissural, and subcortical white matter pathways in 2854 scans (**SI Table 1**).

For each individual, we quantified variability across three complementary dimensions: spatial variability, capturing differences in pathway location and population overlap; microstructural variability, derived from diffusion tensor and neurite-based models [42, 43], [32]); and macrostructural variability, reflecting pathway volume and geometric properties.

These measurements were then used to assess three sources of variation: differences across pathways within individuals, differences across individuals within the population, and changes in variability across developmental and ageing trajectories. Finally, we examined the functional relevance of structural variability by systematically testing associations between pathway variability and individual differences in cognitive, motor, sensory, emotional, and personality traits (eight behavioral and neuropsychological assessments).

All asymmetries, lifespan changes, and interindividual differences reported are statistically significant after correction for multiple comparisons (false discovery rate, FDR < 0.05).

### Spatial variability follows a deep-to-superficial gradient across the brain and lifespan

**Figure 2:**
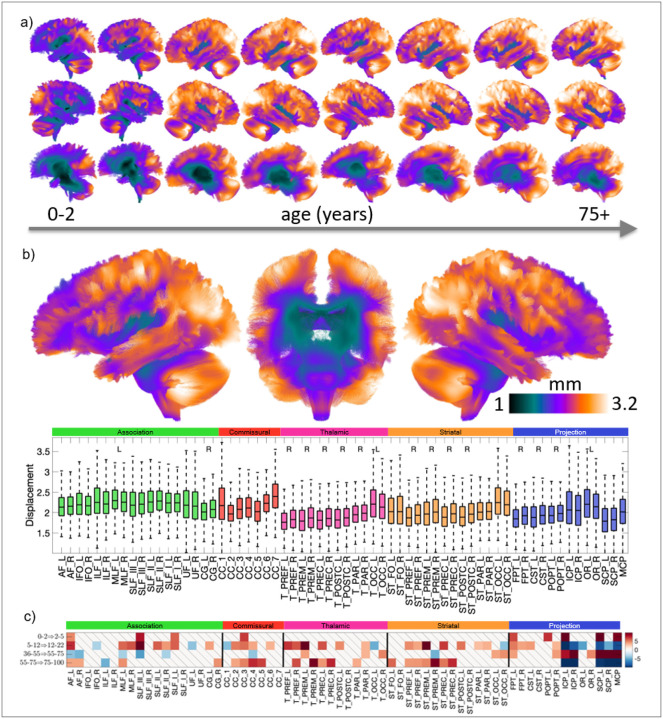
Spatial variability (displacement) across the brain, across bundles, and across the lifespan. (a) Streamlines are colored by average displacement for each cohort. (b) Results from the HCP Young Adult population (age 22–36), where displacements are shown for each bundle, and statistically significant L/R asymmetry is noted. (c) Change in displacement across the lifespan is shown from one cohort to another, for all bundles. “\” indicates no statistically significant change across the lifespan. Statistical tests were controlled by the false discovery rate [109] at 0.05 to determine significance.

Spatial variability across the white matter follows a clear deep-to-superficial gradient, as shown by the average displacement required to align individuals to a common template (Figure 2a,b). Across all age cohorts, variability is lowest in deep white matter structures – including the core of the CST, cingulum, and brainstem – and increases systematically towards the cortical periphery, within the sulci, and the superficial white matter. Increased variability is also observed in primary cortices, including the occipital white matter, motor areas, sensory areas, and auditory areas in the superior temporal gyrus (Figure 2). Lifespan effects are evident, with spatial variability being least pronounced in infancy and increasing throughout development and aging.

Analysis of bundle-specific displacements corroborates these results (Figure 2c). Although displacements are not confined to individual pathways, systematic trends emerge. Association and commissural pathways show larger average displacements than thalamic and projection fibres, displacements increase along an anterior–posterior axis, and most pathways exhibit statistically significant rightward asymmetry.

Changes in spatial variability across the lifespan (Figure 2c) mirror these qualitative patterns. Variability increases modestly in infancy (0–5 years), shows widespread increases during childhood and adolescence (5–22 years), and remains comparatively stable in early adulthood (22–36 years). From mid-adulthood onward (36+ years), spatial variability increases consistently across most pathways, with the notable exception of the cerebellar peduncles.

**Figure 3:**
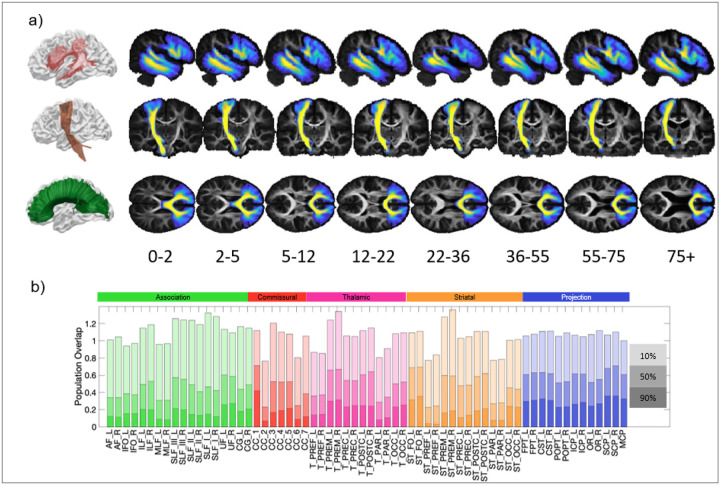
Spatial variability of bundles across the population. (a) Probabilistic maps of the AF left, CST left, and CC_2 are shown in template space, for all cohorts. (b) Normalised population overlap (for young adult data) is shown at 10%, 50%, and 90% overlap, for all 64 pathways.

To quantify inter-individual consistency in pathway location, we generated probabilistic population maps (Figure 3a). These maps provide converging evidence for the deep-to-superficial gradient: across all pathways, spatial overlap is highest in the deep white matter core and decreases towards cortical endpoints. However, this consistency is restricted to a limited portion of each tract (Figure 3b). Regions shared by 90% of participants account for only 10–30% of the average tract volume, whereas regions shared by 50% of the population span approximately 30–70%. Consistent with the displacement analysis, projection and commissural pathways—particularly thalamic and striatal connections to sensorimotor cortices—exhibit the highest population overlap.

### Microstructural variability is pathway-specific and follows a convergence–divergence trajectory

**Figure 4:**
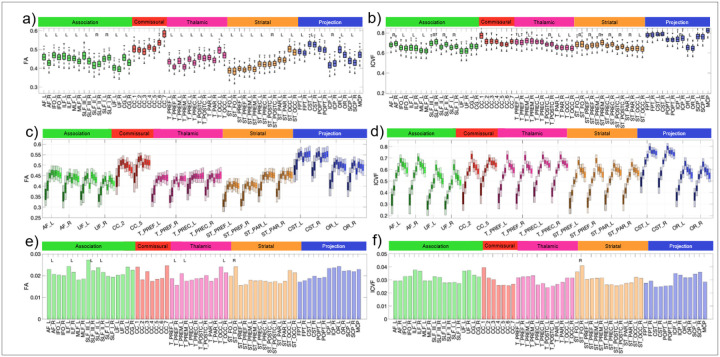
Microstructural variability across the brain (a,b), across the lifespan (c,d), and across the population (e,ft). (a,b) For the young adult dataset, microstructural measures of FA and ICVF are shown for 64 pathways, where statistically significant hemispheric asymmetry is noted as R/L. (c,d) Similar plots are shown across all cohorts where development to aging is colored from light to darker hues. (e,f) variation across a population is shown as bar plots of standard deviation across measures.

White matter microstructure varies considerably across pathways, with distinct profiles for association, commissural, and projection tracts, as well as widespread hemispheric asymmetries (Figure 4a,b). For example, fractional anisotropy (FA) shows anterior-to-posterior increases in commissural and projection (thalamic, striatal) pathways, whereas intracranial volume fraction (ICVF) exhibits an opposing gradient, decreasing from anterior-to-posterior regions. Inter-individual variability also differs by pathway type, with association tracts showing the greatest population-level variability in FA and ICVF (Figure 4e,f).

**Figure 5:**
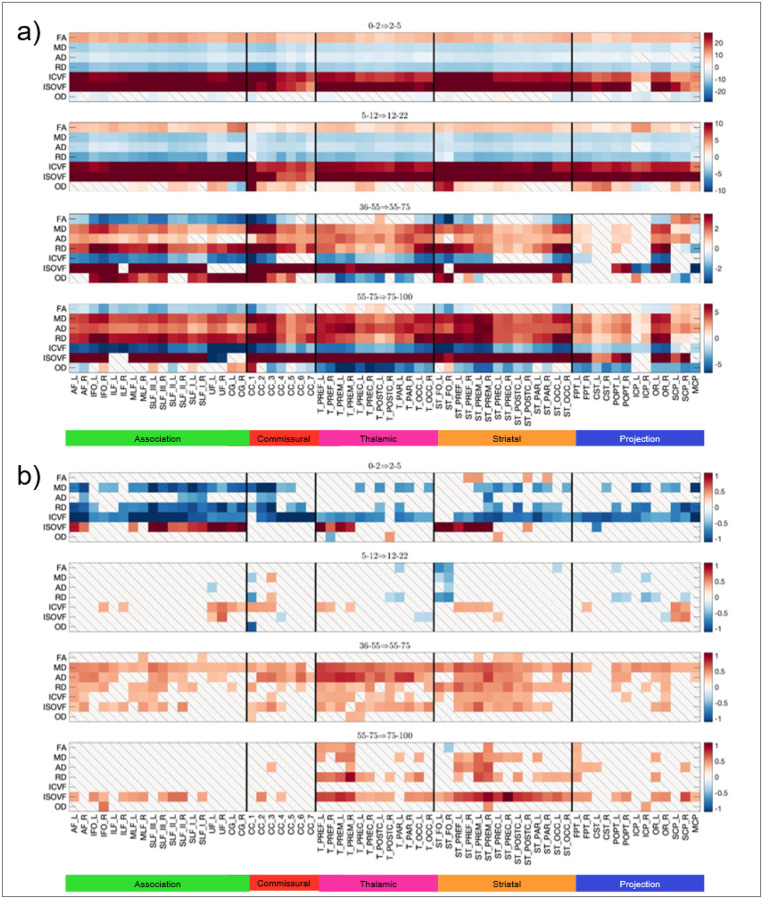
Change in mean microstructural measures (a) and variability of microstructural measures (b) across the lifespan. For all microstructural features, and all pathways, the precent change of the mean (a) or log-ratio of variance (b) is shown (i.e., ratio 0.5, or decrease in variance, is transformed to −.69; a ratio of 2, or increase in variance, is transformed to +0.69). Results are shown for infancy, development, middle adulthood, and late adulthood. “\” indicates no statistically significant change across the lifespan. Statistical tests were controlled by the false discovery rate [109] at 0.05 to determine significance.

Across the lifespan, microstructural features follow well-defined developmental trajectories. During infancy and development, mean FA and ICVF increase while diffusivity measures decrease, consistent with progressive myelination and axonal packing (Figure 4c,d; Figure 5a). These trends reverse in later life, with decreases in FA and ICVF and increases in diffusivity, reflecting age-related microstructural degradation.

Crucially, microstructural variability itself follows a distinct convergence-divergence pattern across the lifespan (Figure 5b). Inter-individual variability decreases during infancy, particularly in association pathways, and remains relatively low throughout development. From middle adulthood onward, however, variability increases markedly—especially for diffusivity-based measures and ICVF—indicating progressive divergence of microstructural trajectories across the ageing population.

### Macrostructural variability scales with tract size and shows distinct lifespan patterns

**Figure 6:**
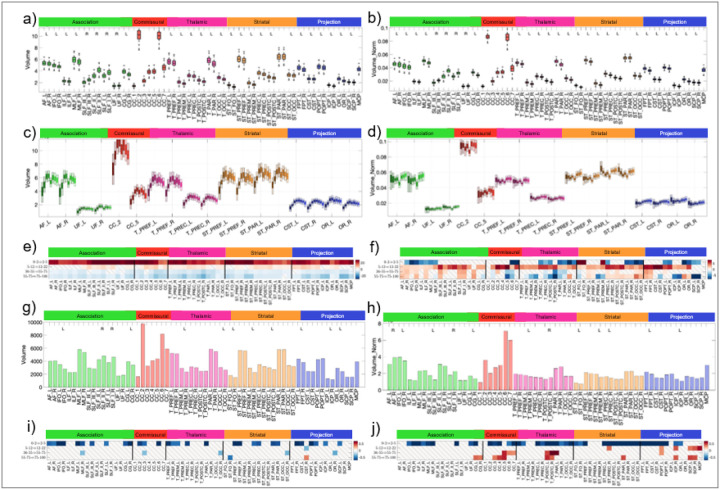
Macrostructural variability across the brain (a,b), across the lifespan (c-f), and across the population (g-j). (a,b) For the young adult dataset, macrostructural features of volume (a) and volume normalised to TBV (b) are shown for 64 pathways, where statistically significant hemispheric asymmetry is noted as R/L. (c,d) Plots are shown across all cohorts where development to aging is colored from light to darker hues. Change in mean macrostructural measures across the lifespan is colored as a percent change of the mean for all ages and pathways (e,f). (g,h) Variation across a population is shown as bar plots of standard deviation across measures. (i,j) Change in variance across the lifespan is colored as the log-ratio of variance (i.e., ratio 0.5, or decrease in variance, is transformed to −.69; a ratio of 2, or increase in variance, is transformed to +0.69). All statistical tests (both asymmetry and variation across lifespan/population were controlled by the false discovery rate [109] at 0.05 to determine significance. “\” indicates no statistical significance for a given pathway.

We investigated macrostructural variability, focusing on pathway volume (Figure 6). Mean tract volumes differed by over an order of magnitude, ranging from the largest segments of the corpus callosum to the smallest long-range association pathways (Figure 6a). Across the lifespan, absolute tract volumes showed a characteristic pattern of rapid increases during development, a plateau in young adulthood, and a decline in aging (Figure 6c,e). When normalised to total brain volume, however, distinct and tracts-pecific trajectories emerged (Figure 6d,f). Notably, in late life, the normalised volumes of many association pathways were preserved, whereas commissural and thalamic pathways show disproportionate reductions.

Across the population, macrostrucutral variability follows two organising principles. First, variability scaled with tract size: the largest pathways exhibited the greatest absolute inter-individual variation in volume (Figure 6a). Second, variability itself showed a distinct lifespan profile. Inter-individual variability in tract volume significantly decreases during infancy, then remained relatively stable from childhood through late adulthood, with few further changes (Figure 6i,j). In addition, inter-individual variability was itself lateralised: for many pathways, the magnitude of volumetric variance differed significantly between hemispheres, indicating asymmetric constraints on macrostructural variability across the population (Figure 6g,h).

### Structural variability predicts individual differences in behaviour

**Figure 7:**
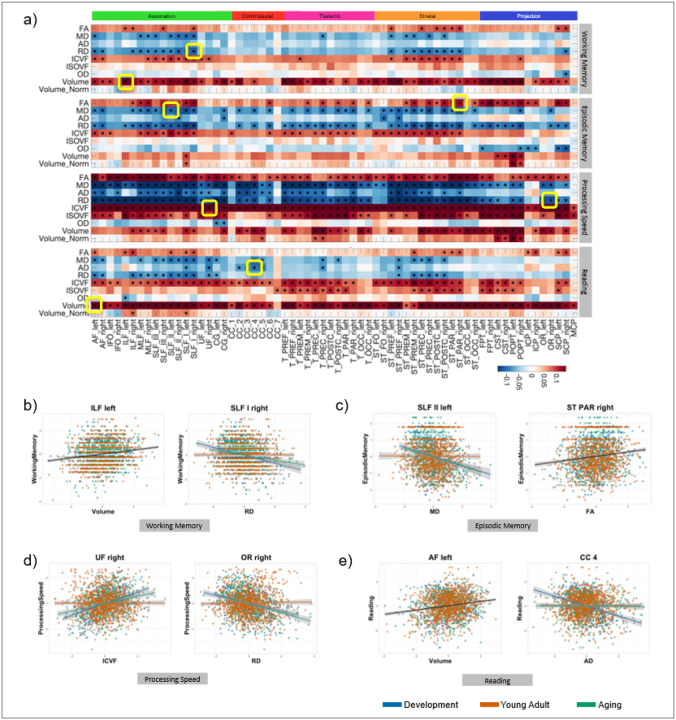
Variability drives structure–function relationships across the brain and lifespan. Variability in white matter features is associated with individual differences in cognitive performance. For four representative behavioral traits (from top to bottom: working memory, episodic memory, processing speed, and reading) the standardized beta coefficients reflecting the association between each tract-level white matter feature and behavioral score are shown (rows: microstructural and macrostructural features; columns: pathways). All maps use consistent contrast scaling for cross-behavior comparisons. Asterisks indicate statistically significant associations after correction for multiple comparisons. For each behavior, exemplar pathway-feature combinations are highlighted and plotted, showing age-normalised data across cohorts. Statistically significant trends are shown as linear fits: black lines denote consistent effects across cohorts, while colored lines indicate cohort-specific associations.

Associations between white matter features and four representative cognitive domains - working memory, episodic memory, processing speed, and reading - are summarized in Figure 7. Three organising principles emerged.

First, structure-function relationships were pathway-specific. Some cognitive functions, such as working memory, showed selective associations with a limited set of tracts, whereas others, including processing speed, were linked to a broad and more distributed structural substrate. This pattern reflects a many-to-one structural functional relationship whereby individual cognitive domains are supported by variability across multiple white matter pathways. For example, working memory was associated with microstructural and macrostructural features of the superior longitudinal and inferior longitudinal fasciculi (SLF, ILF), processing speed with pathways of the internal capsule and brainstem (including the corticospinal and pontine pathways), and reading with the arcuate fasciculus (AF), superior longitudinal fasciculus (SLF), and inferior longitudinal fasciculus (ILF).

Second, associations depended on the specific structural features examined. Microstructural metrics (FA, diffusivity, ICVF) exhibited strong and widespread relationships with behavioural-cognitive performance. In contrast, macrostructural measures showed more nuanced patterns: absolute tract volume showed robust positive correlations across many domains, while volume normalised to total brain size revealed more localized, pathway-specific associations.

Third, the strength of these structure-function relationships was modulated by age. Associations between white-matter variability and behavioural performance were consistently strongest in the developmental cohort and attenuated in young adulthood and ageing. This pattern, evident across multiple analyses (Figure 7), points to a heightened sensitivity of cognitive variability to white matter structure during periods of rapid brain maturation.

Third, the strength of these structure-function relationships was modulated by age. Associations between white-matter variability and behavioural performance were consistently strongest in the developmental cohort and attenuated in young adulthood and ageing. This pattern, evident across multiple analyses (Figure 7), points to a heightened sensitivity of cognitive variability to white matter structure during periods of rapid brain maturation.

## Discussion

In this work, we characterise neurovariability [3] in human white matter - defined as individual differences in the spatial, microstructural, and macrostructural properties of white matter pathways - across the brain, across individuals, and across the lifespan. By mapping 64 long-range association, projection, and commissural bundles in over 2,800 individuals from birth to adulthood, we move beyond the statistical average to reveal how structured anatomical variation underpins human individuality. Our results demonstrate that white matter variability is not random but follows a systemic spatial gradient. Spatial variability follows a deep-to-superficial gradient, microstructural and macrostructural variability exhibit pathway-specific and developmentally dynamic trajectories, and inter-individual differences follow a characteristic convergence–divergence pattern, with increasing anatomical heterogeneity in ageing. Importantly, this variability is functionally meaningful: differences in white-matter architecture predict individual differences in cognitive performance in a manner that depends on pathway, structural feature, and lifespan stage.

Together, these findings challenge the view of neuroanatomical variability as methodological noise or statistical confound. Instead, they establish structured variability as a central and interpretable dimension of brain organisation, defining the anatomical degrees of freedom through which human cognition, development, and ageing are individualised.

### Spatial variability: evolution and development

A primary finding of this study is that spatial variability in white matter architecture is structured rather than random, following a deep-to-superficial gradient, where the core white matter is highly consistent across individuals, and the variability increases systematically toward the cortical periphery. This pattern replicates prior work in human and non-human primates [7] and extends it across the lifespan, suggesting a fundamental “rule” of brain wiring. Deep structures (brainstem, internal capsule, thalami projections) show the greatest spatial consistency, whereas terminal branches of long-range association pathways show the highest variability near gyral crowns and sulcal depths [44]. This spatial gradient reflects the interplay of developmental constraints and evolutionary history, and is well-explained by and consistent with the dual origin theory of the neocortex [45].

This gradient aligns with the dual-origin theory of neocortical evolution, in which the neocortex expands from paraolfactory and parahippocampal allocortical rings with progressively newer association territories added over evolutionary time [46]. The most spatially conserved pathways in our data preferentially link phylogenetically older, developmentally early maturing systems under strong genetic constraint, supporting stable sensorimotor function [47]. In contrast, superficial white matter – including U-fibres and endpoints of association tracts – link the more recently evolved association cortices. These tracts mature later, are more sensitive to experiential shaping, supporting flexible, higher-order functions that are the hallmark of human evolution [48]. The developmental subplate plays a key role in this variability as an embryonic layer that guides thalamocortical and corticocortical axons [49], its evolutionary ancient components scaffold conserved deep connections, while newer additions enable complex cortico-cortical wiring. Our findings also replicate the increased variability near primary cortical regions, including occipital lobes, motor, and sensory areas. This aligns with cytoarchitectonic mapping [50–52], and surface anatomical observations [53], and highlights particularly variable regions such as the parietal cortex, the parieto-occipital sulcus, and rostral prefrontal cortex.

Together, the spatial variability provides insight into both development (early-maturing, conserved connections vs. late-maturing, plastic ones) and evolution (deep, ancient systems vs. recently expanded association cortices), connecting the observed variability to how individual brains are constructed and diversified.

Our probabilistic maps provide a complementary view of the same principle and underscore an important practical implication for atlas-based inference. Across pathways, overlap is high in the deep core but drops substantially towards cortical endpoints [8, 54]. Quantitatively, the region shared by 90% of individuals constitutes only ~10–30% of a tract’s average volume, indicating that a relatively small “common core” is truly conserved in standard space. This suggests that atlas-based analyses are likely to be most spatially reliable in deep tract segments, whereas conclusions that depend on cortical terminations will be more sensitive to individual anatomy (and, potentially, to tractography and registration uncertainty) [55–58]. More broadly, these results motivate approaches that treat terminal anatomy as a meaningful locus of individual variation rather than a nuisance to be averaged away.

### Microstructural Variability

White matter microstructure reflects the biological processes that shape brain circuits across the lifespan. Our results demonstrate that these processes follow a convergence-divergence trajectory: in early life, microstructural variability is reduced as brains converge on a common developmental blueprint, while in later life variability increases as individual differences accumulate and aging trajectories diverge.

During infancy and development, increases in fractional anisotropy (FA) and intracranial volume fraction (ICVF), together with decreases in diffusivities, reflect the maturation of myelination and axonal packing [21]. This sequence closely mirrors Flechsig’s myelogenetic principle, described over a century ago [59], where primary sensory and motor areas myelinate first, followed by association areas, and finally higher-order cortices extending into adolescence [23]. Our findings provide population-level evidence that distinct classes of tracts mature at different rates within this hierarchical program, showing both different microstructural magnitudes and different developmental trajectories. Consistent with this, inter-individual variability decreases during infancy and remains low through development, supporting the concept of **developmental constraint** [60], whereby early wiring is under strong genetic control to ensure consistent establishment of fundamental circuits.

This early convergence stands in contrast to marked later-life population divergence. From middle adulthood onward, inter-individual variability increases substantially, particularly in diffusivity measures (MD), indicating increasing heterogenous ageing trajectories. This divergence likely reflects the combined influence of genetic risk, lifestyle, and resilience factors. The frameworks of brain reserve, cognitive reserve, and brain maintenance [61] provide a useful theoretical framework for this divergence, capturing differences in baseline neurobiological capital, resistance to age-related structural change, and compensatory cognitive strategies, respectively [62]. The widening variance we observe in aging represents the anatomical signature of these mechanisms. Consistent with prior work [19, 63, 64], diffusivity measures appear especially sensitive to these divergent processes, acting as potentially robust biomarkers of heterogeneous aging.

Beyond these lifespan dynamics, microstructural variability is strongly pathway-specific. Different bundles exhibit distinct microstructural profiles, consistent with known variations in neurite density, axon calibre, and myelination across association, commissural, and projection pathways [23–28]. All diffusion-derived microstructural measures showed significant differences across pathways, with systematic distinctions between association, commissural, and projection systems, and anterior–posterior gradients particularly evident in commissural and subcortical pathways. These findings underscore that microstructural variability is not a global property of the white matter but is tightly coupled to pathway identity and function.

Microstructural asymmetry further emerged as a consistent organising feature of white-matter architecture [36, 65–68]. We observed robust hemispheric differences across multiple pathways, including leftward FA asymmetries in the arcuate fasciculus, cingulum, and corticospinal tract, alongside rightward asymmetries in specific branches of the superior longitudinal fasciculus. These patterns roughly align with established functional specialisations for language, motor control, and visuospatial processing [69]. Importantly, the use of multicompartment models revealed that asymmetries in neurite density (ICVF) were more heterogeneous and not always captured by FA alone, underscoring that different microstructural metrics index distinct biological aspects of hemispheric organisation.

Together, these findings position microstructural variability as a constrained yet flexible substrate of brain organisation - highly regulated during development, increasingly permissive in ageing, and central to the anatomical degrees of freedom through which individualised cognitive trajectories emerge.

### Macrostructural Variability

Beyond micro-scale features, individuals also differ in the macrostructural anatomy of their white matter pathways. Our findings show that this variability follows a small set of clear organisational principles. First, macrostructural variability scales with size: larger tracts, such as the corpus callosum and corticospinal tract, show greater absolute variability in volume across the population. Pathways comprising more fibres or spanning longer distances inherently allow more degrees of freedom for inter-individual variation, resulting in larger absolute variance [70]. Second, macrostructural variability follows a distinct lifespan trajectory. During infancy, tract volumes increased rapidly while inter-individual variability often decreases, suggesting strong developmental constraints or convergence toward a common macro-anatomy. After early development, rank-order differences in tract size between individuals remain stable from childhood through adulthood. This pattern suggests that the gross geometry of a tract is largely determined by early developmental programs, creating a stable anatomical scaffold, whereas *microstructure* remains more modifiable by experience and aging [21].

Recent comparative work further supports this interpretation of macrostructural stability emerging from early developmental constraints [71]. Across primate species, large-scale neuroanatomical features show strong continuity with brain volume, independent of phylogenetic distance, suggesting that geometric and mechanical constraints play a central role in shaping macrostructure. Within this framework, certain properties - such as overall tract size and large-scale organisation - are established early and remain remarkably stable, while inter-individual differences arise through differential expansion within these constraints. This view aligns with our observation that macrostructural rank-order differences in tract volume are largely fixed after development, providing a stable anatomical scaffold upon which microstructural plasticity and functional specialisation can unfold.

At the same time, macrostructural asymmetries are widespread. Nearly all major tracts showed volumetric differences between hemispheres, most often favouring the left hemisphere, with consistent exceptions in the uncinate fasciculus and superior longitudinal fasciculus branches (SLF I–III). These patterns replicate prior findings and align with functional specialisation, including rightward volumetric asymmetry in SLF III [8, 72] and in the uncinate fasciculus [11, 73], which have been linked to visuospatial [67] and socio-emotional processing [74]. Mixed or nonsignificant asymmetry effects reported for SLF I and II in smaller samples likely reflect limited statistical power rather than true absence of lateralisation [8, 72]. Together, these findings provide complementary evidence that functional specialization is reflected not only in microstructural but also in the physical size of white matter pathways.

Importantly, we also observed that variability itself is asymmetric: for many pathways, inter-individual variance in tract volume is greater in the left hemisphere than in the right. This suggests that the hemisphere most specialised for higher-order cognition may also permit greater anatomical diversification, consistent with the idea that lateralised systems support individualisation by allowing expanded structural degrees of freedom. At the same time, this pattern should be interpreted in the context of behavioural sampling: many commonly used neuropsychological assessments are language-based or implicitly rely on language processing, thereby disproportionately probing left-hemispheric systems. Such task-space biases have been shown to influence apparent structure–function relationships and hemispheric effects (e.g. [75, 76]) and may amplify left-hemisphere sensitivity to inter-individual variation. Together, these considerations suggest that leftward variance asymmetry likely reflects a combination of genuine biological specialisation and the functional domains most frequently sampled in human cognitive testing.

When macrostructural measures are considered relative to total brain volume, additional lifespan-specific patterns emerge. In late aging, commissural, projection, and cerebellar tracts tend to show disproportionate volume decline, whereas many association pathways and the optic radiations are relatively preserved. This heterogeneity likely reflects differences in ongoing functional demand, plastic potential, and vulnerability across systems. Higher-order associative pathways may retain structural integrity through continued cognitive engagement, while primary visual pathways may benefit from early maturation and lifelong stability, rendering them relatively resistant to age-related atrophy compared to integrative or subcortical circuits.

Finally, macrostructural size has implications for function. Larger tracts generally conferred a performance advantage across multiple cognitive domains, though the pattern became more pathway-specific when normalised to overall brain size. We expand on these structure–function relationships in the following section.

### Structure-Function Relationships of White Matter Variability

Structural variability in white matter is increasingly recognised as a core neurobiological substrate of individual differences in cognition and behaviour [3, 34]. Yet in many neuroimaging studies - particularly group comparisons - inter-individual variability is often treated as noise. Our findings challenge this assumption. Across spatial, microstructural, and macrostructural features, white matter variability robustly associates with individual differences in memory, attention, language, and executive function, demonstrating that variability carries meaningful functional information rather than methodological error. **These differences are likely to be even greater in atypical development or disease, where structural variability can amplify or constrain functional outcomes.**

These structure–function relationships were strongly pathway-specific. Some cognitive domains, such as working memory, were associated with a limited subset of tracts, whereas others - most notably processing speed - were supported by broad, distributed structural substrates. This many-to-one mapping reinforces the view that cognition emerges from large-scale networks rather than single pathways [77–79], with individual differences reflecting coordinated variability across multiple components of the connectome [34].

Distinct structural features contributed in complementary ways. Microstructural measures indexing fibre coherence, myelination, and neurite density showed widespread associations with behavioural performance, reflecting sensitivity to fine-grained biological properties. Macrostructural measures revealed a different pattern: absolute tract volume related broadly to performance, whereas volume normalised to brain size yielded more selective, pathway-specific effects. Together, these results indicate that multiple biological processes operating at different scales jointly shape cognitive variability.

Critically, structure–function relationships were strongest during development, pointing to a period of heightened neuroplasticity in which experience exerts a disproportionate influence on white matter organisation. This pattern aligns with classical and contemporary evidence [80] that learning and environmental input induce measurable structural changes in developing brains. The pronounced coupling observed in development reflects a phase in which white matter variability defines the degrees of freedom available for individualisation within an otherwise constrained developmental architecture.

These findings establish structural variability in white matter as a mechanistic bridge between brain organisation, experience, and individual differences in cognition, with implications for understanding both typical development and vulnerability to neurological or psychiatric disorders.

### Normative models

Our comprehensive mapping of white matter variability provides a principled foundation for normative modelling in human neuroimaging. Traditional case–control comparisons rely on group averages that obscure the substantial biological heterogeneity present within both healthy and clinical populations. In contrast, normative modelling offers an individual-centred framework, analogous to paediatric growth charts, in which brain structure is evaluated relative to age-appropriate population distributions[81]. By quantifying how white matter architecture varies across pathways, features, and the lifespan, our results define the expected range of anatomical variability against which individual brains can be meaningfully contextualised. This enables the identification of personalised deviation profiles - revealing *where*, *how*, and *to what extent* an individual departs from normative developmental or ageing trajectories. Such deviation maps offer increased sensitivity for detecting early neurodevelopmental vulnerability, tracking subtle neurodegenerative change, and characterising heterogeneity within diagnostic categories, thereby supporting the transition towards precision neuroscience and personalised clinical inference [82–84].

### Limitations and Future Work

Several limitations and boundary conditions should be acknowledged. First, spatial variability is inherently dependent on aligning scans to a template. Our templates were chosen based on meaningful neurobiological transitions across the lifespan [81, 85], enabling consistent comparison of developmental stages within a harmonised acquisition framework. While alternative template choices are possible, there is currently no consensus on what constitutes an optimal population reference, particularly across wide age ranges. Second, this registration and alignment is more challenging at the brain’s periphery, leading to increased variance in these areas. However, this is exactly the variance we hope to describe through the spatial variability measures, capturing meaningful population-level spatial differences that our displacement measures aim to quantify. Third, all features, both microstructure and macrostructure are heavily dependent upon acquisition choices and sensitive to preprocessing [86–90]. While it is helpful to mitigate this through a harmonized acquisition, this is not feasible for all centres and studies, and in particular, historical datasets. Instead, harmonization is necessary, albeit unlikely post hoc, to enable comparisons across different acquisition conditions. Moreover, some measures still await validation in the literature – particularly macrostructural features (e.g. volume, lengths, and surface areas). Finally, the current study was limited to long-range association, projection, and commissural pathways. Future work should extend this framework to include validated superficial white matter systems [91–95], brainstem pathways, and to more diverse populations and clinical cohorts.

## Conclusion

Using over 2,800 diffusion MRI scans spanning the human lifespan, we provide a comprehensive, multi-scale characterisation of normative white matter variability across 64 long-range pathways. We show that variability in white matter architecture is not random but anatomically structured, developmentally dynamic, and laterally patterned, following systematic gradients across pathways, hemispheres, and age. Across individuals, white matter variability decreases during early development and increases in later life, particularly within association pathways, revealing ageing as a period of growing neuroanatomical divergence. Crucially, this variability is functionally meaningful: individual differences in white matter anatomy predict cognitive and behavioural performance across multiple domains, establishing structural variability as a substrate of cognitive individuality rather than methodological noise. Together, these findings reposition neuroanatomical variability as a core dimension of brain organisation—one that reflects the balance between developmental constraint and lifelong plasticity. By providing a normative, lifespan-informed framework for mapping variability at scale, this work lays the groundwork for precision approaches to brain development, ageing, and disease.

## Methods

### Datasets

The data used in this study come from three branches of the Human Connectome Project [38], which aims to map the structural connections and circuits of the brain and their relationships to behaviour by acquiring high-quality magnetic resonance images. We used diffusion MRI data from the Baby Connectome Project [39] (Howell et al., 2019), the Human Connectome Project Development (HCP-D), the Human Connectome Project Young Adult (HCP-YA), and the Human Connectome Project Aging (HCP-A) study. This manuscript will refer to these as Infant, Development, Young Adult, and Aging cohorts, respectively.

The Infant cohort was composed of 259 participants and 543 imaging sessions (1–5 sessions per participant) aged between 0 and 5 years. The Development cohort was composed of 652 participants aged 5 to 21 years. The Young Adult cohort was composed of 1206 participants aged 21 to 35 years. The Aging cohort was composed of 722 participants aged 35 to 100 years. After quality assurance (removal of datasets with excessive diffusion arti-facts, failure of tractography, the pooled dataset was composed of 2854 imaging sessions and spanned the ages of 1 week to100 years old. Data are summarized in Figure 1.

The diffusion MRI acquisitions were slightly different for each dataset and tailored towards the population under investigation. For Development and Aging cohorts, a multi-shell diffusion scheme was used, with b-values of 1500 and 3000 s/mm^2^, sampled with 93 and 92 directions, respectively (24 b=0). The in-plane resolution was 1.5 mm, with a slice thickness of 1.5 mm. For the Young Adult cohort, the minimally preprocessed data [96] from Human Connectome Projects (Q1-Q4 release, 2015) were acquired at Washington University in Saint Louis and the University of Minnesota [38] using a multi-shell diffusion scheme, with b-values of 1000, 2000, and 3000 s/mm^2^, sampled with 90 directions each (18 b=0). The in-plane resolution was 1.25 mm, with a slice thickness of 1.25 mm. The Infant cohort typically used a 6-shell sampling scheme with b-values of 500, 1000, 1500, 2000, 2500, and 3000 s/mm^2^, sampled with 9, 12, 17, 24, 34, and 48 directions, respectively (14 b = 0). Depending on compliance, however, a protocol matched to the Development cohort was sometimes also used for the Infant cohort (see [39] for discussion on acquisition). The in-plane resolution was 1.5 mm, with a slice thickness of 1.5 mm. For all diffusion data, susceptibility, motion, and eddy current corrections were performed using TOPUP and EDDY algorithms from the FSL package following the minimally preprocessed HCP pipeline [96].

### Data Processing

We grouped data into eight time periods roughly aligned with developmental steps to inform a more fine-grained parcellation of meaningful transitions in the lifespan [81, 85]. We defined early infancy (0–2), late infancy (2–5), childhood (5–12), adolescence (12–21), young adulthood (21–35), middle adulthood (35–55), older adulthood (55–75), and late life (75+). Details of the age ranges, mean age, and sample size are shown in Figure 1 in table and histogram formats. We note that cohorts were not mixed between time periods, as differences in acquisition may lead to differences in quantitative metrics.

For every session, sets of white matter pathways were virtually dissected using the TractSeg [41] automatic white matter bundle segmentation algorithm. TractSeg was based on convolutional neural networks and performed bundle-specific tractography based on a field of estimated fibre orientations [41]. From these outputs, we selected 64 white matter bundles spanning six categories: association (intra-hemispheric cortico-cortical connections), commissural (inter-hemispheric fibres), thalamic (projections between thalamus and cortex), striatal (cortico-striatal projections), projection and cerebellar (long-range connections between cortex and subcortical structures, brainstem, spinal cord, or sensory relay nuclei, and connections involving the cerebellum). A full list of pathways, acronyms, and classifications is provided in the Supplementary Material.

### Spatial variability

Our aim was to quantify spatial variability as measures of similarity or differences in the spatial location of white matter and specific pathways. Following [7], our first measure of spatial variability was an average measure of displacement, or deformation, required to align all participants’ data in a standard space. To do this, we created cohort-specific unbiased FOD templates (i.e., 8 templates) using MRTrix3 software [97].

Briefly, for each cohort, 25 randomly selected (but sex-matched) volunteers were selected for template creation. The cohort averaged white matter response function was derived by averaging response functions (using *dwi2response dhollander* algorithm [98, 99]), images were up-sampled to 1.25mm isotropic (mrgrid command), fibre orientation distributions were reconstructed (using *dwi2fod msmt_csd* algorithm [100]), and joint bias field correction and intensity normalization peformed (mtnormalise command [101]). Finally, the study-specific unbiased FOD templates were created using the *population_template* command and default parameters. Tractography on the template FODs was performed for visualization purposes (using *tckgen iFOD2* algorithm [102]). The FOD image from all participants was registered to the corresponding FOD template (mrregister command). From the warp fields and following [7], we quantified every individuals’s deformation, i.e., a voxel-wise displacement to warp/align the individual to the template. Thus, population-averaged displacement (for each cohort) was our first measure of spatial variability. It described the shift of each point in an individual’s image to get to its corresponding point in the template image. We also calculated the Log of the Jacobian of the transformation field, which provides insight into local volume changes (local scale, rotation, shear) resulting from the displacement. We show these in the supplementary material.

To measure how consistently a pathway occupies the same location across individuals, we calculated a ‘normalised population overlap’ index. This index tells us what fraction of a pathway’s average total volume is shared by a specific percentage of the population. For example, we calculated this index at the 10%, 50%, and 90% levels. The 90% overlap index, specifically, represents the proportion of the pathway’s average volume that is found in the same spatial location for at least 90% of the individuals studied. A higher index value indicates greater spatial consistency (less variability) for that portion of the pathway across the population, while a lower value signifies more spatial variability. All bundles for all participants were transformed to template space using the warp fields derived above. Once in standard space, population-based atlases were created following methods previously used to create tractography atlases [54, 103, 104]. For each pathway, binarized maps were summed and set to a voxel-wise population probabilistic map between 0 and 100%. Here, a value of 75% in a voxel represents that the pathway exists in that location in 75% of the population. Our normalize population overlap index is calculated at 10%, 50%, and 90% as the total volume of >10%, >50%, or >90% of population overlap divided by the average volume of that pathway across the population. For example, to calculate the “90% normalised population overlap” of the AF_left, we would calculate the total volume where 90% of individuals overlap and normalize by the average AF_left volume.

### Microstructural measures

Our second aim was to quantify and investigate the microstructural variability of each pathway. For each pathway, we derived four microstructural-sensitive parameters from Diffusion Tensor Imaging (DTI) [42, 43] and three microstructural-sensitive parameters from the Neurite Orientation Distribution and Density Imaging (NODDI) model.

DTI captures the anisotropic diffusion of water molecules in brain tissues as a (covariance matrix of a) 3D Gaussian distribution. From DTI, scalar indices of diffusivity were derived, including axial (AD), radial (RD), and mean diffusivities (MD), and indices of orientation anisotropy can be derived, including fractional anisotropy (FA) [43]. These indices are sensitive to several features of tissue microstructure, including axonal packaging, myelination, axon diameters, and geometric configuration (undulating fibres, fanning fibres, crossing fibres) [30]. All DTI indices were derived from only data with b<1500 s/mm^2^ (see Table 1) (in line with best practices for tensor fitting [105]).

NODDI was developed to increase specificity to multiple tissue compartments [32]. Hence, this multicompartment model leads to measures of intracellular volume fraction (ICVF) as a measure of neurite density, isotropic volume fraction (ISOVF) (as a measure of free water), and neurite orientation dispersion (OD) as a measure of geometrical dispersion of neurites ranging from 0 (isotropic) to 1 (perfectly aligned). NODDI indices were derived from all b-values used in the acquisition.

For DTI and NODDI, the average value of each index was extracted for each pathway using the SCILPY toolbox (*scil_bundle_mean_std.py*) using default parameters (specify here).

### Macrostructural measures

We considered “macrostructure” as geometrical features of pathways using shape descriptors of volumes, areas, and lengths of the bundles. Recent work has shown macrostructural trends across the lifespan [106] and increased predictive power of cognitive performance when incorporating shape-based features of pathways [107]. To derive macrostructural features of each pathway, we used the SCILPY toolbox (scil_bundle_shape_measures.py), which resulted in 12 macrostructural features (including tract volume, span, curl, diameter, surface area, elongation, area of endpoints, volume of endpoints; see [108] for details of all features). In this work, we investigated the variability of total tract volume and tract volume normalised to total brain volume (a normalization common in cortical analysis, also see Forkel et al. Brain 2014). Other macrostructural measures are provided as supplementary material.

### Sources of variability

We investigated three primary sources of variation. The first variability measure, was intraindividual variability or “variation across the brain”, i.e., what is the typical variation across pathways? To answer this, we showed boxplots of a given feature (e.g. FA), grouped by pathway type (i.e. association, commissural, thalamic, striatal, projection, cerebellar) and (when possible) ordered along an anterior-to-posterior gradient. This allowed visualization of trends or differences across pathways. We additionally performed a Wilcoxon signed rank test (a nonparametric test for two populations when the observations are paired) to test for left/right asymmetries in each feature and each pathway. Due to multiple comparisons, all statistical tests were controlled by the false discovery rate [109] at 0.05 to determine significance.

The second variability measure was the “variation across the population”, i.e., what is the typical variation in some feature across the cohort? To answer this, we showed bar plots of the standard deviation of a given feature, grouped by pathway type and ordered anterior-to-posterior to visualize trends across pathways. We performed a Wilcoxon signed rank test, controlled by the false discovery rate, to test for hemispherical asymmetries.

The third source of variability was the “variation across the lifespan”, i.e., does the mean value (e.g., FA or volume) change across the lifespan? In addition to average changes, we were also interested in the variation around the mean and scrutinized if the standard deviation changes across the lifespan. We display boxplots of the mean values across each cohort for selected pathways to visualize trends across the lifespan. We perform additional tests to ask whether there are statistically significant changes in the mean or variance from one cohort to another by using a two-sample t-test to test for equal means or two-sample F-test to test for equal variance. We note that because of differences in acquisition and scanners (see Table 1), there are expected biases in both microstructure and macrostructure [89, 90]. Comparisons are, therefore, only made when comparing similar datasets. This allowed us to test for differences in infancy (from the 0–2 to 2–5 cohorts), differences in development (from 5–12 to 12–22 cohorts), differences in middle adulthood (36–55 to 55–75 cohorts), and differences in late adulthood (55–75 to 75–100 cohorts).

### Brain Cognition and Behavior Relationships

To link the observed white matter variability to function, we investigated the relationship between pathway-specific features and cognitive domains. This analysis was performed on participants from the Development, Young Adult, and Aging cohorts. We selected eight behavioural measures spanning multiple cognitive and emotional domains that were available across the datasets: Episodic Memory (Picture Sequence Memory), Executive Function/Inhibition (Flanker Task), Card Sorting, Language/Reading Decoding (Oral Reading Recognition), Processing Speed (Pattern Completion Processing Speed), Working Memory (List Sorting), Emotion Recognition (Penn Emotion Recognition Test), and Self-regulation/Impulsivity (Delay Discounting).

This analysis aimed to answer two primary questions: (1) To what extent does white matter variability explain variance in cognitive-behavioural processes across individuals? (2) Does the association between white matter features and behaviour change across the lifespan?

To prepare the data for statistical testing, we first normalised the structural features. For each of the nine features (FA, MD, AD, RD, ICVF, ISOVF, OD, Volume, Volume_Norm) across all 64 pathways, values were z-scored within each of the three datasets (Development, Young Adult, Aging). Outliers, defined as data points greater than four standard deviations from the mean of that feature within that dataset, were removed. A similar normalization and outlier removal procedure was applied to each of the eight behavioural scores.

To test our questions, we fit a multiple linear regression model for each pathway, feature, and behavior combination:

$$\text{Behavior}\sim \beta_{0}+\beta_{1}(\text{Feature})+\beta_{2}(\text{Dataset})+\beta_{3}(\text{Feature}\times \text{Dataset})+\beta_{4}(\text{Age})+\beta_{5}(\text{Sex})+\epsilon$$

The model assessed the association between a pathway’s feature and a behavioural score (B_1_) while controlling for covariates. We tested for a statistical interaction between the pathway feature and dataset (as a proxy for lifespan epoch), where significant interaction term (Feature×Dataset) would indicate that the relationship between the white matter feature and behaviour is significantly different across the Development, Young Adult, and Aging cohorts.

Given the large number of statistical tests performed (64 pathways × 9 features = 576 tests per behavioural measure), we employed a strict Bonferroni correction to control for multiple comparisons. A relationship was considered statistically significant if its p-value was below a corrected threshold of p<0.05/576. Significant associations were visualized using scatterplots to illustrate the nature and direction of the brain-behaviour relationships.

## Supplementary Material

Supplementary Files

This is a list of supplementary files associated with this preprint. Click to download.

- SupplementaryTable1.docx

## Data Availability

Templates for each lifespan epoch are available (10.5281/zenodo.18318460) which include population-averaged fractional anisotropy, fibre orientation distributions, streamlines, and probabilistic maps for each fibre pathway. The data used in this study come from the Human Connectome Project, which aims to map the structural connections and circuits of the brain and their relationships to behaviour by acquiring high-quality magnetic resonance images. We used diffusion MRI data from the Baby Connectome Project (HCPBaby), the Human Connectome Project Development (HCPD) study, the Human Connectome Project Young Adult (HCP) study, and the Human Connectome Project Aging (HCPA) study. Data from the Human Connectome Project – Aging (HCPA) dataset are available upon request from https://www.humanconnectome.org/study/hcp-lifespan-aging. Data from the Lifespan Baby Connectome Project (HCPBaby) dataset are available upon request https://www.humanconnectome.org/study/lifespan-baby-connectome-project. Data from the Human Connectome Project – Development (HCPD) dataset are available upon request from https://www.humanconnectome.org/study/hcp-lifespan-development. Data from the Human Connectome Project – Young Adult (HCP) dataset are freely available for download from https://www.humanconnectome.org/study/hcp-young-adult.
